# Respective effects of phlebotomy losses and erythropoietin treatment on the need for blood transfusion in very premature infants

**DOI:** 10.1186/1471-2431-13-176

**Published:** 2013-10-28

**Authors:** Odile Becquet, Delphine Guyot, Philippe Kuo, Françoise Pawlotsky, Marianne Besnard, Micheline Papouin, Alexandre Lapillonne

**Affiliations:** 1 Department of Neonatology, APHP Necker Hospital, Paris, France; 2 Department of Neonatology, Territorial Hospital of Tahiti, Papeete, French Polynesia; 3 Paris Descartes University, Paris, France

**Keywords:** Erythropoietin, Anemia of prematurity, Erythrocyte transfusion, Blood loss

## Abstract

**Background:**

The benefit to risk ratio of the treatment with erythropoietin (EPO) as a means of limiting the number of transfusions in very preterm infants during hospitalization, seems to be modest since the adoption of restrictive transfusion criteria and of policy limiting phlebotomy losses. We therefore aim to evaluate the factors associated with the number of late blood transfusion in very preterm infants in a unit where the routine use of EPO has been discontinued.

**Methods:**

A comparative “before-after” study was carried out in premature infants born before 32 weeks postmenstrual age (PMA), over a period of one year before (EPO group) and one year after (non-EPO group) the discontinuation of EPO therapy.

**Results:**

A total of 48 infants were included in the study (EPO=21; non-EPO=27). The number of infants transfused after the 15 day of life (D15) and the number of transfusions per infant after D15 were not significantly different between the two groups. In a multivariate analysis, the gestational age and the volume of blood drawn off during the first month of life significantly influenced the need for transfusions after the 15th day of life, independently of the treatment with EPO. The hemoglobin levels measured at different times of hospitalization (median postnatal age: 16, 33 and 67 days) were not significantly different between the two groups.

**Conclusions:**

Our study shows that the discontinuation of EPO did not change the number of late transfusions. Even when a policy limiting phlebotomy losses is used, blood loss is an important and independent risk factor for late transfusion of very preterm infants.

## Background

The anemia of premature infants is more severe and more prolonged than of term neonates. Below a certain threshold, this anemia becomes pathologic as it no longer permits a tissue oxygenation adequate for growth and development, and then, a blood transfusion is required. Since infants born prematurely display low erythropoietin (EPO) plasma levels and a retarded increase in its secretion, the use of recombinant EPO to limit the number of transfusions in premature infants has been proposed since a pilot study published in the 90’s [1]. The controlled randomized trials which were then published showed that the use of EPO in premature neonates significantly reduces the number of transfusions and the volume of blood transfused [2-6]. These studies also highlighted the facts that very broad and liberal transfusion criteria were used [3] and that the quantities of blood drawn off could be responsible of important blood losses [7]. The studies published since 2000 indicate that the effects of EPO treatment on the requirement for blood transfusions are moderate if more strict transfusion criteria and policy to limit phlebotomy losses are applied [8-12]. Furthermore, they showed that EPO does not reduce the need for transfusion within the first 15 days of life [13,14] on account of the delayed action of the hormone [15].

In 2006, the Cochrane collaboration published three meta-analyses [14,16,17]. The first showed that administration of EPO from the 8th day of life afforded a reduction in the volume of blood transfused of 7 mL/kg/infant and a diminution of 0.78 transfusions per infant. Conversely, the use of EPO did not diminish the risk of transfusion as there was no significant reduction in the number of donors [16]. The second indicated that EPO therapy started within the first 7 days of life permitted a decrease in the volume of blood transfused of 6 mL/kg/infant, a diminution of 0.33 transfusions per infant and a significant decrease in the number of donors. This study revealed, on the other hand, a significant increase in the incidence of retinopathy of stage ≥3 [17]. Finally, the third meta-analysis showed that the number of transfusions and the volume of blood transfused were similar whether EPO was administered early (before 8th day of life) or late [14]. Since these successive analyses, more strict transfusion criteria have been progressively adopted by neonatal intensive care units (NICUs) in France and other countries, and the indications for treatment with EPO have been progressively restricted [18].

For about 10 years, we have implemented in our NICU a policy of conservative blood management, and a protocol including strict blood transfusion criteria. In view of the above data showing a modest impact of EPO treatment on transfusion requirements, together with the potentially severe side effects [19] and the fact that the procedure is painful for the infant [20], it was decided in our neonatology unit to suspend EPO therapy in premature newborns as of 1st August 2010. The objective of the present study was to evaluate the effects of this policy change on late transfusion requirements (i.e., after 15 days of life; (≥D15)) and on the evolution of hemoglobin levels during hospitalization.

## Methods

### Study design

A “before-after” study was carried out in the NICU of the Territorial Hospital Center of French Polynesia during two consecutive years before and after the discontinuation of EPO therapy: from 01/08/2009 to 31/07/2010, i.e., one year before the arrest of EPO treatment (treated group) and from 01/08/2010 to 31/07/2011, i.e., one year after the arrest of treatment (non-treated group). The data were retrieved from the medical records.

### Inclusion and exclusion criteria

All premature infants born at a postmenstrual age (PMA) < 32 weeks and a birth weight ≤ 1500 g during the study periods were included. The exclusion criteria were infants suffering from a congenital malformation or a hemolytic disease (i.e., blood group incompatibilities and G6PD or pyruvate kinase deficiencies), and those who had required surgery.

### Patient care protocols

The infants belonging to the treated group received EPO (Epoetin beta, NeoRecormon®, ROCHE, France) from the first week of life (1st injection between D3 and D7), at a dose of 250 IU/kg three times a week subcutaneously for 6 weeks, i.e., a total of 18 injections.

The service has a policy of conservative blood management and a single donor for a given patient. Blood samples were drawn into tubes clearly indicating the quantity of blood necessary or were capillary samples. The quantity of blood required for blood cultures (0.5 mL) was measured in a syringe before being injected into the blood culture flask. Samples were analyzed by micro-methods and these methods were not modified between the two periods of the study. The samples were transferred rapidly to the laboratory by means of a pneumatic tube system. A limited number of physicians were responsible for the prescription of biological tests: the clinician in charge of the unit and the duty doctor. Particular attention was paid to the frequency and grouping of the blood tests. Finally, the unit protocol includes giving back the blood drawn on an umbilical line before the actual blood sample is obtained.

Enteral feeding was introduced progressively and as a function of the digestive tolerance. All infants were fed with pasteurized human milk until they reach 32 weeks corrected age, and then fed with either mother’s milk enriched with a fortifier (Eoprotine®, MILUMEL, France) at a final concentration of 3% or a preterm formula (Pregallia®, DANONE, France). The feeding protocol was not modified between the two periods of the study.

All the infants received an enteral iron supplement after the 7th day of life as soon as the total enteral intake reached ≥100 mL/kg/d. The initial dose was 1.4 mg/kg/d and was increased stepwise by 1.4 mg/kg/d every 48 h, according to the digestive tolerance, up to a target dose of 6.8 mg/kg/d (Sodium feredetate, Ferrostrane®, TEOFARMA, Italy). All the infants of the study were likewise given a folic acid supplement for one month, at a dose of 1.25 mg/d orally, as soon as the enteral intake exceeded 100 mL/kg/d. The protocol for iron and folic acid supplementation underwent no modifications between the two periods of the study.

The indications for transfusion were the following protocol throughout the 2 study periods: 1) hemoglobin <12 g/dL if the infant was less than 48 h old, required respiratory support with FiO2 ≥40% or presented pulmonary arterial hypertension; 2) hemoglobin <9 g/dL in the case of respiratory support with Fi02 <40%, poor weight gain, episodes of hypoventilation, severe associated pathology or surgery; 3) hemoglobin <8 g/dL in other cases. The volume of blood delivered in each transfusion was 15 mL/kg.

### Data collection

The epidemiological, clinical and biological data for each infant included were recovered from the medical records by one person (O.B.). Intrauterine growth retardation (IUGR) was defined as a birth weight of less than the 10th percentile for the gestational age on reference curves [21]. The achievement of respiratory autonomy was defined as spontaneous respiration in ambient air without tracheal or nasal support. Postnatal steroid therapy was defined as systemic therapy targeting the lungs and administered ≥15 days of life. Severe cerebral lesions were defined as intraventricular hemorrhage (IVH) of a grade ≥3 of the Papile classification [22] or as cystic periventricular leukomalacia. The presence of retinopathy of any stage, of a nosocomial infection suspected or confirmed by clinical, biological or bacteriological data, or of necrotizing enterocolitis of stage ≥3 of the Bell classification was likewise retrieved.

Hemoglobin levels were measured at different times of hospitalization: during the first 24 hours of life (Hb at day 0), between the 14th and 21st days of life (Hb at 2 to 3 wks), between the 28th and 42nd days of life (Hb at 1mo) and between the 6th week of life and discharge from hospital or at the death of the infant (Hb at discharge). The theoretical volume of blood drawn off for biological tests during the first 30 days of life was noted and recorded as a function of the birth weight.

The number of infants transfused and the number of transfusions per infant were retrieved for the period from the day of birth to discharge from hospital or death. Owing to the geographic situation of the hospital, no secondary transfers were performed. Transfusions were categorized as early when they took place <15 days of life and late ≥15 days of life.

### Ethical information

This study was conducted according to the guidelines of the Declaration of Helsinki. According to French legislation, neither ethical approval nor informed consent was required for this non-interventional, retrospective cohort study.

### Statistical analyses

Statistical analyses were performed using Minitab 13.3® software (Minitab Inc., Pennsylvania, USA). Qualitative variables were expressed as percentages and quantitative variables as medians and extremes. The variables “gestational age at birth” and “birth weight” were divided into the following classes: <28 weeks, 28–29.9 weeks and 30–32 weeks for the gestational age and <1000 g and ≥1000 g for the birth weight. Discontinuous data were compared with the Fisher exact test and continuous data with the Mann–Whitney U test. To determine the factors that affect the need for transfusion after the 15th day of life, the factors presenting a degree of significance of p ≤ 0.1 in the univariate analysis (i.e., intrauterine growth retardation, gender, gestational age) were included in a binary logistic regression model together with the volume of phlebotomy losses and the treatment with EPO. The relations between these factors were expressed as odds ratio with a 95% confidence interval. Tests were considered to be significant for a p value of less than 0.05.

## Results

### Description of the study groups

All eligible infants (n=48) were included in the study: 21 in the treated group and 27 in the non-treated group. None were excluded because of postnatal transfer since the unit is geographically very far from any other level II ou level III units (i.e., no other neonatal unit in the island of Tahiti). The clinical characteristics of the children are presented in Table 1. The two groups were comparable for all criteria except gender (p=0.01), the incidence of IUGR (p=0.04) and severe neurological lesions (p=0.02). Two children died during the study on D43 and D54 respectively; both belonged to the treated group. The hemoglobin at birth, the number of early transfusions and the volume of blood drawn off during the first month of life were comparable (Table 2).

**Table 1:** Clinical characteristics of the newborns included in the study

	**Treated group (n= 21)**	**Non treated group (n= 27)**	**Total (n=48)**	**p**
Gender*				
boy	14 (67 %)	8 (30 %)	22 (46 %)	0.01
Gestationnal age (week) **	28.1 [26.1-31.8]	29.3 [25.6-31.8]	28.6 [25.6-31.8]	0.1
Birth weight (g)	1100 [802- 1428]	1110 [666- 1496]	1105 [666- 1496]	0.7
Birth length (cm)	36.5 [30.0- 40.8]	37 [31- 43]	37 [30- 43]	0.3
Birth head circumference (cm)	26.5 [22.0- 29.5]	26 [22.5- 28.5]	26 [22- 29.5]	0.4
Birth weight < 1000 g	7 (33 %)	8 (30 %)	15 (31 %)	0.8
Intrauterine growth retardation	3 (14 %)	11 (40 %)	14 (29 %)	0.04
Apgar score				
1 mn	7 [2- 10]	6 [3- 10]	6.5 [2- 10]	0.3
5 mn	9 [5- 10]	9 [7- 10]	9 [5- 10]	0.3
10 mn	10 [8- 10]	10 [8- 10]	10 [8- 10]	0.9
Duration of hospitalization (d)	67 [40 - 102]	63.5 [37-96]	65 [37 - 102]	0.3
Respiratory autonomy (d)	40 [4 - 94]	37 [0 - 88]	37.5 [0 -94]	0.2
Severe cerebral lesions	4 (19 %)	0 (0%)	4 (8 %)	0.02
Post natal steroid therapy	3 (14 %)	3 (11 %)	6 (12 %)	0.7
Retinopathy	2 (9 %)	3 (11 %)	5 (10 %)	0.8
Nosocomial infection	10 (48 %)	6 (22 %)	16 (33 %)	0.06
Necrotizing enterocolitis	3 (14 %)	2 (7 %)	5 (10.4)	0.4

* n (%); **median [Min-Max].

**Table 2:** Hematological data of the newborns with and without erythropoietin therapy

	**Treated group (n=21)**	**Non treated group (n=27)**	**Total (n=48)**	**p**
Volume of phlebotomy losses (ml/kg)*	16.8 [6.9 - 49.4]	14.5 [7.6 - 50.0]	15.1 [6.9 - 50.0]	0.7
Start of iron supplementation (age in days)	22 [13 - 51]	20 [5 - 38]	20.5 [5 - 51]	0.5
Transfusions before day 15 of life				
Nb of infants transfused <D15**	9 (43 %)	5 (18 %)	14 (29 %)	0.07
Transfusions per infant <D15	0 [0 - 2]	0 [0 - 1]	0 [0 - 2]	0.9
Transfusion after day 15 of life				
Nb of infants transfused ≥D15	7 (33 %)	9 (33 %)	16 (33 %)	1.00
Transfusions per infant ≥D15	0 [0 - 3]	0 [0-2]	0 [0 - 3]	0.6
Day 0 of life				
Haemoglobin (g/dL)	15.2 [11.9-17.7]	15.9 [13.1-18.9]	15.5 [11.9-18.9]	0.2
2-3 wks				
Haemoglobin (g/dl)	11.4 [8.1-14.8]	11.3 [6.7-12.9]	11 [6.7 - 14.8]	0.9
Age at sampling (d)	15 [14 - 21]	16 [14 - 21]	16 [14 - 21]	0.3
1 month of life				
Haemoglobin (g/dl)	9.9 [8.4 -12.8]	10.0 [8.1- 12.9]	10 [8.1 - 12.9]	0.7
Age at sampling (d)	34 [29- 39]	32 [28- 39 ]	33 [28 - 39]	0.4
Discharge				
Haemoglobin (g/dl)	10.4 [7.7 -12.4]	9.6 [8.4- 11.7]	9.6 [7.7 - 12.4]	0.3
	*n=13*	*n= 21*	*n=34*	
Age at sampling (d)	72 [43 - 99]	65 [46 - 93]	67 [43 - 99]	0.9

*median [Min-Max], ** n (%).

### Effects of the EPO treatment

The number of infants transfused after D15 was identical in the two groups (Table 2). The number of transfusions per infant after D15 (mean ± SD = 0.5 ± 0.9 in the treated group and 0.4 ± 0.6 in the non-treated group) was not significantly different nor was the volume of blood transfusion of the transfused infants (mean ± SD = 23.6 ± 11.8 mL/kg in the treated group and 18.3 ± 6.6 mL/kg in the non-treated group).

In the binary logistic regression model, only the variables “gestational age” and “volume of phlebotomy losses” were significantly and independently associated with the need for transfusion after 15 days of life, whereas treatment with EPO was not significant (Table 3). The levels of hemoglobin at different times of hospitalization were not significantly different between the two groups (Table 2).

**Table 3:** Influence of different factors on the need for transfusion after 15 days of life

**Variables**	**Odds ratio**	**95% IC**	**p**
Gestational age at birth*	0.18	0.03-0.96	0.044
Intrauterine growth retardation	0.63	0.06-6.94	0.704
Gender (boy)	0.22	0.03-1.92	0.171
Volume of phlebotomy losses (mL/kg/d)	1.17	1.01-1.36	0.032
Treatment with erythropoietin	0.15	0.01-1.60	0.118

*The variable “gestational age at birth” was divided into the following classes: <28 weeks, 28–29.9 weeks and 30–32 weeks.

## Discussion

Our study shows that the discontinuation of EPO therapy did not significantly modify the number of infants transfused, the number of transfusions per infant after D15 or the hemoglobin levels after the 15th day of life in infants born very preterm. These results are concordant with those of the literature, which indicate that the clinical effects of EPO are limited or absent when restrictive transfusion criteria are employed [8,9,12,23,24]. Our work also confirms that the use of restrictive transfusion criteria is effective to limit the number of infants transfused [2,3,5,12,13,24-30]. Based on the results of the literature, the French authorities issued guidelines questioning the need for EPO when policies of restrictive transfusion criteria and a single donor for repeated transfusions were applied [18].

Cell transfusion practices vary widely among practicing neonatologists throughout the world [31]. Some studies have shown that it is equally efficacious to employ a conservative transfusion protocol as to use early administration of EPO in premature infants of gestational age <30 weeks and/or birth weight <1500 g [24]. Others have demonstrated an absence of any increase in mortality, duration of hospitalization or occurrence of severe cerebral lesions, apnea, retinopathy or bronchopulmonary dysplasia when a conservative transfusion policy was employed [23,24]. However, although most authors have a tendency to use a restrictive guidelines, the use of a restrictive or liberal guidelines for red blood cell transfusion in preterm infants, remain a controversial issue since their impact on the neurosensorial and neurocognitive development through infanthood and childhood is inconsistent [26,32-35].

Our study shows that the volume of phlebotomy losses is an independent factor significantly associated with the need for late transfusion. Previous study have shown that considerable phlebotomy losses are a risk factor associated with the need for early transfusion in very preterm infants. For example, it has been shown in infants not treated with EPO, that the volume of blood losses during the first 7 days of life is a significant predictive factor for transfusion over the first 7 days of life [36]. In a randomized controlled trial testing a bedside blood analyzer, blood transfusions administered to extremely low birth weight infants were reduced by decreasing laboratory phlebotomy losses [37]. Our study adds to the literature by the fact that the volume of phlebotomy losses is associated with the need for late transfusion even if an effective blood sparing protocol is applied. Indeed, our blood saving protocol is one of the most efficient published to date since the volume of phlebotomy losses during the first month of life was ~0.5 mL/kg/d in the two groups, which is a figure lower than most of the values published (Table 4).

**Table 4:** Comparison of the volumes of phlebotomy losses in preterm infants published in the literature

**References**	**Year of publication**	**Population studied**	**Number of infants**	**Estimated losses (ml/kg/d)**
[5]	1993	27-33 wks	24	0.6
[2]	1993	< 33 wks	51	0.4
[3]	1994	750 - 1500 g	241	0.7
[36]	1997	≤ 1500 g	47	3.3
[25]	1999	≤ 32 wks	100	0.6
[26]	2000	< 32 SA and < 1250g	114	0.9
[12]	2001	< 1000 g	172	1.8
		1000–1250 g		0.8
[13]	2002	< 1000 g	219	0.7
[37]	2005	500 - 1000 g	93	5
[27]	2006	450 - 800 g	40	0.6
[24]	2006	< 30 wks or > 1500 g	50	2
[28]	2008	1000 - 1750 g and 28–34 wks	40	0.8
[29]	2011	≤ 1250 g and < 30 wks	30	0.4
[6]	2013	500 – 1250 g	99	0.8
Present study		<32 weeks and < 1500 g	47	0.5

Our work presents several limitations. The retrospective nature of the data collection imposes the limits inherent to this type of study. However, the number of infants transfused, the number of transfusions per infant and the evolution of hemoglobin levels were unlikely to be affected by their retrospective retrieval due to the existence of a transfusion record in all the medical files and the electronic recording of all biological results. On the other hand, there is probably some uncertainty concerning the actual volume of blood drawn off and this could be underestimated [7], although it is unlikely that the error induced would be different between the two groups since the sampling procedure did not change during the period of the study. We could not assess the impact of the discontinuation of EPO therapy on reticulocytosis as the number of infants in whom this test was performed was too low, even though it is recognized that this parameter is affected by EPO [9,38]. The small number of patients in each group is a weakness of the study and makes the demonstration of the direct effect of the intervention (arrest of EPO) more uncertain. We could nevertheless calculate that the number of children included in the study allowed one to test the hypothesis of a doubling of the risk of transfusion with a power of 80% and an alpha risk of 0.05. Our work shows that a gestational age of <28 weeks is an independent risk factor for late transfusions and hence it would be appropriate to specifically test the effects of EPO in this subgroup of extremely premature infants. Finally, our study was not able to determine the role of delayed cord clamping on the need for late blood transfusion since the procedure was not used in our perinatal department during the study periods.

## Conclusion

In conclusion, this study showed that the number of late transfusions had not increased significantly one year after the discontinuation of EPO. In units where policies of conservative transfusion and single donors are applied, it would seem reasonable to discontinue the use of early EPO treatment for very preterm infants for which the administration procedure is painful, the large scale clinical efficacy is modest and the absence of side effects does not appear to be fully established. In the area of conservative transfusion policy and blood sparing, we found that phlebotomy losses remained an important risk factor for late transfusion.

## Abbreviations

EPO: Erythropoietin; D: Days; PMA: Postmenstrual age.

## Competing interests

The authors have no competing interests to declare.

## Authors’ contributions

OB: Provided clinical and scientific along the project, in particular for definition of objectives, patient inclusion and non-inclusion criteria validation, patient recruitment, acquisition of data, interpretation of results, and choice of concepts to be measured; reviewed critically the manuscript; approved the final version of the manuscript. DG: Made substantial contributions to acquisition of data and interpretation of data; have been involved in revising the manuscript critically for important intellectual content; and have given final approval of the version to be published. PK: Made substantial contributions to acquisition of data and interpretation of data; have been involved in revising the manuscript critically for important intellectual content; and have given final approval of the version to be published. FP: Made substantial contributions to acquisition of data and interpretation of data; have been involved in revising the manuscript critically for important intellectual content; and have given final approval of the version to be published. MB: Made substantial contributions to acquisition of data and interpretation of data; have been involved in revising the manuscript critically for important intellectual content; and have given final approval of the version to be published. MP: Made substantial contributions to acquisition of data and interpretation of data; have been involved in revising the manuscript critically for important intellectual content; and have given final approval of the version to be published. AL: Provided clinical and scientific expertise on EPO along the project, in particular for definition of objectives, patient inclusion and non-inclusion criteria validation, patient recruitment, interpretation of results, and choice of concepts to be measured; reviewed critically the manuscript; approved the final version of the manuscript. All authors read and approved the final manuscript.

## Pre-publication history

The pre-publication history for this paper can be accessed here:

http://www.biomedcentral.com/1471-2431/13/176/prepub
